# The Importance of the Plasma Membrane in Atherogenesis

**DOI:** 10.3390/membranes12111036

**Published:** 2022-10-24

**Authors:** Stanislav Kotlyarov, Anna Kotlyarova

**Affiliations:** 1 Department of Nursing, Ryazan State Medical University, 390026 Ryazan, Russia; 2 Department of Pharmacy Management and Economics, Ryazan State Medical University, 390026 Ryazan, Russia

**Keywords:** atherosclerosis, cardiovascular disease, plasma membranes, lipid rafts, membrane proteins

## Abstract

Atherosclerotic cardiovascular diseases are an important medical problem due to their high prevalence, impact on quality of life and prognosis. The pathogenesis of atherosclerosis is an urgent medical and social problem, the solution of which may improve the quality of diagnosis and treatment of patients. Atherosclerosis is a complex chain of events, which proceeds over many years and in which many cells in the bloodstream and the vascular wall are involved. A growing body of evidence suggests that there are complex, closely linked molecular mechanisms that occur in the plasma membranes of cells involved in atherogenesis. Lipid transport, innate immune system receptor function, and hemodynamic regulation are linked to plasma membranes and their biophysical properties. A better understanding of these interrelationships will improve diagnostic quality and treatment efficacy.

## 1. Introduction

Atherosclerotic cardiovascular disease is a global problem of modern medicine. The increasing prevalence, medical and social consequences of atherosclerosis emphasize the importance of the problem and raise awareness of the need to address it [1]. Coronary heart disease, ischemic stroke and peripheral artery disease occupy an important place in the structure of population morbidity and carry a heavy economic and social burden [2,3].

Atherosclerosis is an actively studied problem. In the complicated history of the study of atherosclerosis, many theories of its pathogenesis have been proposed. The lipid theory is widely known and underlies today’s therapeutic approaches in both the treatment and prevention of atherosclerosis, which is achieved through the correction of lipid disorders. Indeed, dyslipidemia, metabolic syndrome and obesity are among the leading problems of modern society, which are associated with low physical activity and poor diet. Importantly, lipid accumulation in the vascular wall is one of the first imaging events in atherogenesis. Macrophages, key participants in the innate immune system, accumulate lipids by turning into foam cells.

At the same time, there is a growing body of evidence that supports a role for the innate immune system and inflammation in atherogenesis [4]. In addition, lipid mediators derived from fatty acids are actively involved in inflammation in atherosclerosis. Leukotrienes play an important role in atherogenesis; whereas specialized pro-resolving mediators, in contrast, are involved in the resolution of inflammation.

An important conceptual advance in vascular biology has been the understanding of the complex role of endothelial cells, whose function is associated with the regulation of blood flow and the behavior of other cells in the vascular wall [5]. There is a growing body of data demonstrating the important role of endothelial cell mechanobiology and how this function is linked to the role of the plasma membrane [6]. The data obtained to date have greatly expanded our understanding of the structure and function of the plasma membrane and have also increased our understanding of the clinical significance of their impairment. Thus, the purpose of this review is to discuss the role of the cellular plasma membrane and the significance of changes in its structure and function in atherogenesis.

## 2. Function of Plasma Membranes and Membrane Proteins

The structure and function of cellular plasma membranes have been the subject of numerous studies, the results of which have improved and systematized our understanding that the plasma membrane is not only a complex multicomponent structure that serves the function of delimiting the living cell from the surrounding space, but is also a platform on which various biological processes are organized, providing many cell functions [7,8].

According to modern concepts, the plasma membrane is a lipid bilayer, in which proteins are integrated. At the same time, the lipid bilayer is represented by various types of lipids, distributed asymmetrically between the two bilayer leaflets. An important component of plasma membranes is cholesterol, which, due to its chemical structure, participates in the lateral organization of the lipid bilayer of the plasma membrane, largely determining its biophysical properties and functions [9]. The location of the cholesterol molecule in the plasma membrane is determined by its chemical structure [10]. The cholesterol molecule is oriented with steroid rings parallel to the hydrocarbon chains of membrane phospholipids. The hydroxyl groups of the cholesterol molecule are located between the polar head groups of phospholipids near the lipid-water interface. Cholesterol hydroxyl groups often form hydrogen bonds with oxygen atoms of phosphate and ester groups of lipids. The polycyclic cholesterol ring in this arrangement is located in the thickness of the membrane, where it can reach the depth of C9 to C10 carbon atoms of phospholipid acyl chains [11]. It is important to note that the rigid tetracyclic cholesterol ring has an asymmetric structure, which includes a flat α-surface and a β-surface containing two methyl groups. Sphingolipids usually interact with the α-surface of cholesterol, and transmembrane protein domains interact with the β-surface [12,13]. This structure of the cholesterol molecule allows for a denser packing of lipids, which increases the viscosity of the lipid bilayer of the membrane. Large hydrophilic head groups are known to protect the hydrophobic tails of membrane phospholipids from the external polar environment. At the same time, cholesterol has a smaller hydrophilic head group than phospholipids. In this regard, its presence in the membrane cannot protect the hydrophobic region as effectively as phospholipids, so in the presence of cholesterol, the phospholipid tails become more aligned as they are packed closer together. At the same time, the area they occupy decreases [14]. As a consequence, changes in cholesterol content can influence the main mechanical parameters of the plasma membrane, such as stiffness, elasticity, and resistance to rupture under load [15,16,17,18,19,20]. Thus, an increase in the concentration of cholesterol in the plasma membrane increases the ordering of phospholipid tails, which manifests itself as a decrease in membrane fluidity due to the reduced lateral movement of membrane phospholipids. Moreover, the effect of cholesterol on membrane fluidity varies depending on the depth [11,21,22,23]. Near the surface, cholesterol reduces membrane fluidity, whereas in the center of the membrane, cholesterol increases membrane fluidity because the cholesterol ring reaches a depth approximately equal to the C9 position of the carbon atom in the acyl chains [11]. This may be important for the spatial arrangement of membrane proteins.

It is important to note that cholesterol is unevenly distributed in the plasma membrane, which provides a unique lateral organization of the membrane that involves the simultaneous existence of a lipid ordered phase and a lipid disordered phase [11]. The lipid ordered phase is associated with the so-called lipid rafts, which are involved in many physiological processes [24]. It is assumed that microdomains of the lipid ordered phase, called lipid rafts, float in the disordered phase like «icebergs in the ocean».

Lipid rafts are cholesterol- and sphingolipid-enriched special structures of the cell plasma membrane, which provide many essential cellular functions by acting as dynamic platforms recruiting a number of signaling and transport proteins [25] The concept of lipid rafts was proposed 25 years ago, but still remains a subject of discussions, including not only their structure and function, but also the fact of their existence, due to the complexity of their visualization [26]. Two types of plasma membrane lipid domains are well known: planar lipid rafts enriched with glycosylphosphatidylinositol (GPI) and related proteins, and caveolae, which are microdomains invaginated inside the cell and stabilized by caveolin structural proteins [27]. Caveolin-1 is the main framework protein of caveolae, where it forms oligomeric structures. *Cav1* gene knockout leads to loss of caveolae by cells [28]. It is suggested that Caveolin-1 can directly interact with cholesterol through cholesterol recognition/interaction amino acid consensus (CRAC) domain [29]. Cholesterol depletion leads to a decrease in the number of caveolae; whereas excessive cholesterol saturation leads to a decrease in plasma membrane fluidity and an increase in the number of caveolae [30].

It is assumed that the size of lipid rafts is approximately 10 to 200 nm, while the diameter of the opening of caveolae on the surface of the plasma membrane is about 60–80 nm [31,32,33]. Caveolae are supposed to be more stable than planar lipid rafts and can have different shapes, including flat, tubular, or vesicular [34]. Although caveolae and planar lipid rafts have a certain similarity in lipid composition, the possibility of a mutual transition between them has not been confirmed [35].

Cholesterol is an important component of lipid rafts, making a significant contribution to their structural organization [36]. Due to the polycyclic ring, cholesterol provides a denser packing of lipids in rafts. This increases the viscosity of lipid rafts compared to non-raft domains. Importantly, the function of membrane proteins that reside in lipid rafts may depend on specific lipid-protein interactions [12,37]. Membrane lipids can affect the conformation and function of membrane proteins by altering membrane structure and physical properties. They can change the local thickness of the membrane and the curvature of the lipid bilayer and can alter physical properties of the membrane, including cholesterol levels, phospholipid types, and acyl chain length and movement [38,39]. In addition, cholesterol may be involved in regulating the function of transmembrane proteins through direct interaction of sterol with specific protein sites. Proteins that interact with cholesterol can contain characteristic amino acid sequences that are involved in this interaction. Such sequences include the cholesterol-binding amino acid domain (CRAC), which has been identified in proteins that interact with or are regulated by cholesterol. The CRAC amino acid sequence is characterized by the following set of amino acids: (L/V) − X (1–5) − (Y) − X (1–5) − (R/K) [12]. The CARC motif, which has similar properties in binding to transmembrane proteins, has the reverse amino acid sequence: (R/K)-X (1–5) − (Y/F)-X (1–5) − (L/V) (with X = any amino acid), and tyrosine can be replaced by phenylalanine. It is assumed that the CRAC sequence contributes to a certain extent to the localization of membrane proteins in lipid rafts. However, the mere presence of such a domain does not necessarily indicate the occurrence of specific cholesterol-protein interactions, and experimental data are needed to confirm them.

The data obtained in recent years have significantly expanded our understanding of the structure and function of the cell membrane [40,41]. Of particular interest are the mechanisms of interactions between the cortical actin cytoskeleton and the plasma membrane [42]. According to the concept known as the “fences and pickets model”, it is assumed that the liquid plasma membrane is separated by actin-based membrane skeleton “fences” and anchored transmembrane protein “pickets” [41,43,44]. The actin-based membrane skeleton is located directly on the cytoplasmic surface of the plasma membrane. The actin cytoskeleton, specific to the eukaryotic cell, is thought to be involved in a wide range of cellular processes, from shape determination and phagocytosis to intracellular transport and cytokinesis [45].

The actin cytoskeleton is thought to contribute to the partitioning of the plasma membrane into small compartments, which are involved in the temporary confinement of phospholipids [42,44]. This mechanism ensures lateral segregation of lipids, not only those located in the inner leaflet of the plasma membrane, with which the cortical F-actin cytoskeleton is associated, but also those located in the outer leaflet of the membrane. In addition, the model of pickets of anchored transmembrane proteins suggests that various transmembrane proteins can be anchored and aligned along the membrane skeleton, acting as rows of pickets against the free diffusion of membrane molecules [44]. Importantly, pickets affect both lipid and transmembrane protein trafficking, whereas fences mainly affect only transmembrane proteins. Immobilization of the transmembrane protein attached to the membrane skeleton provides an increase in fluid viscosity around it due to the effects of hydrodynamic friction on the surface of the immobilized protein. This makes it difficult for membrane molecules to pass to the compartment boundaries of the anchored transmembrane proteins aligned along the membrane-skeleton fence [41,43]. This has implications for the redistribution and clustering of receptors and their signaling.

These data and the results of other macrophage studies that the pickets do not need to be permanently attached to the cytoskeleton but can bind to the cortical actin through reversible interactions. In addition, the stability of the cytoskeletal mesh, which creates diffusion-limiting enclosures, can vary greatly, which is characteristic of myeloid cells, for example [46]. Circulating leukocytes at quiescent state have a dense cortical actin cyto-skeleton polymerized with formins. However, when leukocytes cross the endothelium and enter the tissues, actin becomes branched and severed [46]. Such changes may, to some extent, turn off the function of the pickets, facilitating the ability of the receptors to diffuse and form oligomers. Phosphatidylinositol 4,5-bisphosphate (PtdIns (4,5) P2) has an asymmetric distribution in the plasma membrane and is involved in maintaining the polarization of individual actin networks, which is important for immune cells [46]. Thus, the picket-anchored model can explain some functions of immune cells and mechanisms of their regulation.

It is important to note that the model of a fixed picket fence does not exclude the existence of other principles of plasma membrane organization, such as lipid rafts [41].

Thus, the “picket fence model” postulates that lipid diffusion can be confined to the juxtamembrane cortex and, therefore, can participate in the formation of membrane domains. In this case, the organization of the plasma membrane of atherogenic cells probably results from a combination of mechanisms expected in both the “picket-fence” model and the “lipid-raft” model.

Thus, variations in the content of individual lipid components of lipid rafts can lead to changes in the function of membrane proteins. Included in phospholipids, saturated and polyunsaturated fatty acids can differentially affect the structure and function of lipid microdomains [47,48]. In addition, proteins in lipid rafts often have posttranslational modification by fatty acids, such as palmitoylation and myristoylation [47], which contribute to protein localization in lipid rafts [49]. In addition, glycosylphosphatidylinositol (GPI) anchors contribute to the localization of proteins in lipid rafts. S-acylation, often referred to as S-palmitoylation, is one of the key factors controlling the association of proteins with rafts. S-palmitoylation is the attachment of a palmitic acid residue (C16:0) to a protein cysteine residue via a thioether bond [50]. S-palmitoylation affects protein function, including their stability, localization in membranes, and interprotein interactions [51,52]. By increasing membrane affinity, palmitoylation promotes stable protein binding to the membrane [53,54,55]. At the same time, palmitoylation is a reversible process and is carried out by a complex of enzymes [53,56,57].

Analyzing the biophysical principles of the organization of the plasma membrane, it is important to note the asymmetric distribution of lipids between its leaflets [58]. In mammals, the asymmetry of negatively charged phosphatidylserine, which is localized mainly on the inner leaflet of the plasma membrane, is well known [59]. In addition to phosphatidylserine, phosphatidylethanolamine is also found in the inner leaflet of the plasma membrane of many cell types, whereas phosphatidylcholine and sphingomyelin are localized in the outer leaflet. It is known that the asymmetric distribution of phospholipids may be related to their unsaturation. It was found that the cytoplasmic leaflet of the plasma membrane is twice as unsaturated as the exoplasmic leaflet [59]. Given that saturated lipids are more densely packed, they correspond to stiffer and more ordered membranes. In contrast, high levels of unsaturated lipids correspond to relatively liquid, loosely packed membranes. This suggests a difference in the biophysical properties of plasma membrane leaflets, in which the outer leaflet of mammalian plasma membranes may be more tightly packed and ordered than the inner leaflet [59]. This structural asymmetry of lipids is reflected in asymmetric structures of transmembrane protein domains [59]. A growing body of evidence suggests the presence of a complex system of mutual regulation between membrane proteins and the surrounding lipid microenvironment in the plasma membrane [59].

Membrane lipid asymmetry is associated with the action of transport mechanisms and has important biological significance. Membrane localization of phosphatidylserine is a well-known “eat me” signal, due to which it functions as a link between the dying cell and the phagocyte [60].

In addition, phosphatidylserine is an important part of the actin cortex connection to the plasma membrane [61,62]. Data obtained in recent years have suggested that interactions between the cortical actin cytoskeleton and living asymmetric membranes are related to the clustering behavior of anchored GPI proteins on the outer leaflet of the plasma membrane. GPI anchor, which is a complex glycolipid, has long saturated acyl chains such as C16:0 or C18:0. GPI plays an important role in the delivery of the attached membrane protein to the plasma membrane. Although the details of the mechanisms of nanocluster formation remain the subject of debate, the importance of acyl chain interactions between GPI-anchored proteins and phosphatidylserine localized in the inner leaflet of the plasma membrane has been shown to result in the formation of clustered domains on the plasma membrane [61,62]. Such clustering, in accordance with the active composite membrane model, may be related to the dynamic organization of actin into nanoscopic assemblies known as “asters” [63,64]. It is suggested that this is due to myosin activity, which promotes the organization of short dynamic actin filaments into “aster-like” configurations that can stimulate nanoclustering of proteins and lipids of the outer membrane leaflet [61]. Using some adaptor proteins, these actin assemblies can bind phosphatidylserine, which contains long saturated acyl chains that participate in lipid-mediated interactions with GPI-anchored proteins located in the opposite leaflet containing long acyl chains [61,62]. In this case, the chemical composition of the fatty acid chain of the GPI anchor plays an important role in the occurrence of nanoclustering [61].

Thus, phosphatidylserine containing a long acyl chain is essential for interlayer coupling in the plasma membrane [61,62]. Indeed, it has been shown that immobilization of lipids with a long saturated acyl chain can stabilize cholesterol-dependent interlayer interactions, which promote the formation of local domains with liquid-ordered (lo) phase characteristics [62]. The importance of phosphatidylserine is supported by the data that cells unable to produce sufficient amounts of phosphatidylserine due to mutations in the enzymes involved in its biosynthesis lack the ability to create GPI nanoclusters on the plasma membrane, but this ability was restored when phosphatidylserine with a long chain of saturated fatty acids (18:0) was added [61]. Thus, proteins and lipids localized on the outer leaflet of the plasma membrane can also interact with cortical actin through this mechanism [65].

It should be noted that the active composite membrane model describes the dynamic response of glycosylphosphatidylinositol-anchored proteins (GPI-APs) on the outer leaflet as a consequence of internal motion under the action of active actomyosin-dependent stresses [65]. It was shown that at temperatures above the equilibrium segregation of the lipid phase a possible combination of active contractile stresses generated by the cortical actomyosin, transbilayer interactions between phosphatidylserine and GPI/lo lipids and lateral lipid interactions is suggested. This leads to the formation of active emulsion, mesoscale liquid order (lo) domains of the GPI-APs and lipids. These domains, enriched with GPI-APs nanoclusters, are supported by cortical actin activity and interlayer interactions and are characterized by significant lipid order [65]. These data enhance the understanding of the significance of lipid asymmetry, cortical actin, and their dynamic interactions with the biophysical properties of the plasma membrane as well as the localization and function of membrane proteins.

Thus, plasma membranes having a dynamic complex composition are a self-regulating system, which is capable not only of being a site for proteins and their signaling pathways, but also a tool for regulation of their functional activity under the influence of internal and external factors.

## 3. Involvement in the Regulation of the Innate Immune System

The innate immune system relies on a large number of pattern recognition receptors to detect molecular structures associated with damage. Toll-like receptors (TLR) are the most well characterized family of receptors that are involved in the detection of a wide range of intra- and extracellular molecular structures. TLR4 is the most studied toll-like receptor, and its role in atherogenesis is of growing interest, given the importance of macrophages in atherosclerotic lesions. Activation of toll-like receptor signaling can contribute to the development of atherosclerosis through multiple mechanisms. TLR4, TLR2, and MyD88-deficient mice had reduced development of atherosclerosis [66]. TLR4 is expressed at low levels by human endothelial cells in normal arteries, but expression is markedly increased in endothelial cells in the region of atherosclerotic lesions [67].

TLR4 detects lipopolysaccharides (LPS) of the cell wall of Gram-negative bacteria and can localize both on the plasma membrane and in endosomes. TLR4 homodimerization is the initial step in receptor activation [68,69,70]. It is assumed that, upon activation, TLR4 localizes in the lipid rafts of plasma membranes, which may regulate its activity [71]. The possibility of direct interaction of TLR4 with cholesterol in lipid rafts is described. This is due to the presence of CRAC and CARC sequences in the transmembrane domain of TLR4, which can provide a link between cholesterol and the regulation of signal transduction of the receptor [71]. Thus, the regulation of TLR4 activity can be carried out through changes in the cholesterol content in lipid rafts of macrophage plasma membranes. It has been shown that enrichment of free cholesterol in plasma or endosomal membranes of macrophages leads to activation of signal transduction through various TLRs [72]. In addition, LPS and lauric acid induced dimerization and recruitment of TLR4 to lipid rafts. At the same time, docosahexaenoic acid inhibited LPS or lauric acid induced dimerization and recruitment of TLR4 to lipid rafts. Lauric acid was shown to stimulate and docosahexaenoic acid to inhibit the association of TLR4 with MD-2 and downstream adaptor molecules, as well as the activation of NF-κB expression [73]. Docosahexaenoic acid is highly unsaturated, making it sterically incompatible with cholesterol, which can lead to disruption of lipid rafts [74].

It has also been shown that eicosapentaenoic acid can incorporate into lipids in lipid rafts, inhibit their formation and inhibit some signaling pathways [75,76]. Eicosapentaenoic acid has been shown to inhibit T-lymphocyte activation, probably by displacing acylated signaling proteins from membrane lipid rafts [75].

LPS recognition and TLR4-mediated signaling in myeloid cells is facilitated by CD14, which contains a GPI part attached to its C-terminal, which anchors the protein in the outer leaflet of the plasma membrane in lipid rafts [77,78,79]. Once in the plasma membrane, CD14 binds LPS and facilitates the transfer of LPS to the MD-2 protein of the TLR4/MD-2 complex [77,80,81].

It seems interesting that S-palmitoylation also controls TLR signaling. In experiments on the macrophage cell line RAW264.7, exposure to LPS was shown to alter the palmitoyl profile of these cells [82]. LPS was found to trigger S-palmitoylation and activation of phosphatidylinositol 4-kinase type II (PI4KII) β, which generates PI(4)P, leading to inflammatory activation of macrophages [77].

Toll-like receptor-mediated inflammation has been shown to require FASN-dependent palmitoylation of MYD88. Binding of IRAK4 to the MYD88 intermediate domain and subsequent signal activation required MYD88 palmitoylation [83]. Accordingly, blocking S-palmitoylation of MYD88 suppressed TLR-mediated inflammation. In addition, CD36 maintains the intracellular pool of palmitate required for S-palmitoylation of MyD88. Meanwhile, CD36 function is also dependent on S-palmitoylation, highlighting the complexity of the CD36-TLR signaling axis and the important role that S-palmitoylation plays here [84]. S-palmitoylation is necessary for the complete localization of another representative of this receptor group, TLR2, on the cell surface, which affects its interaction with its ligands [85].

Cholesterol crystals are a common element present in atherosclerotic lesions. An increase in the frequency of their formation is observed in atherosclerotic lesions as they progress [86,87]. There is an assumption that cholesterol crystals may appear due to high cholesterol content in cell membranes of the arterial wall, which is accompanied by peroxidation of membrane phospholipids [88]. Increased cholesterol content and the presence of polyunsaturated phospholipids in these membranes together with oxidative stress can lead to the formation of pure bilayer cholesterol domains. With further peroxidation and increased cholesterol, they can break down as cholesterol crystals [88]. Cholesterol crystals activate inflammasomes and induce IL-1β secretion by monocytes and macrophages, thereby stimulating immune responses and initiating inflammation, which can lead to the development of atherosclerosis [88].

Thus, the innate immune system actively involved in atherogenesis may be related to the function of plasma cell membranes, which is a promising topic for further research.

## 4. Participation in Hemodynamic Regulation

Numerous data suggest that disorders of vascular hemodynamics contribute significantly to the development and progression of atherosclerosis. It has been shown that despite the systemic nature of many risk factors, such as dyslipidemia, systemic inflammation and oxidative stress, atherosclerotic lesions do not develop diffusely, but more often localize in the arterial areas characterized by bifurcations, bends and curvatures [89,90,91].

**Figure 1:**
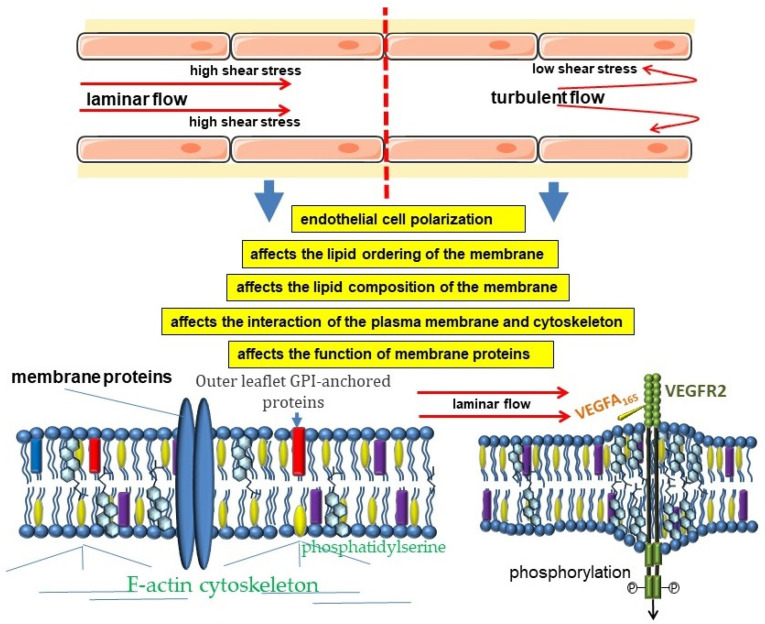
Cross-links of hemodynamic forces and biophysical properties of the plasma membrane of endothelial cells.

The modern concept of vascular hemodynamics suggests an active role of the endothelium in the regulation of blood flow. Endothelial cells have a wide set of mechanisms for detecting changes in the character of blood flow and its regulation through the production of several biologically active substances [92,93,94]. At the same time, the endothelial cells themselves are constantly under the influence of several hemodynamic forces, among which the leading role belongs to the shear stress. The magnitude of shear stress, as well as the nature and rate of its change, are considered to be the key hemodynamic characteristics influencing mechanobiology and endothelial function (Figure 1) [95]. It is believed that high laminar blood flow is characteristic of straight arterial sections, which correlates with resistance to atherosclerosis. On the contrary, the mean shear stress value is lower in areas of curved or branching arteries. In addition, blood flow in these areas undergoes complex changes in direction during the cardiac cycle, which is generally referred to as “disturbed flow” [96]. Such areas are characterized by the development of atherosclerosis.

Endothelial cells are able to actively respond to changes in the nature of blood flow, which allows them to adapt most effectively to the action of hemodynamic forces. Changes in the nature of blood flow can affect the orientation and morphology of endothelial cells [97,98,99,100,101,102,103,104,105,106,107].

When endothelial cells are exposed to hemodynamic forces, the lipid ordering and cholesterol content of the plasma membranes change, which leads to changes in the biophysical properties of the membrane. This, in turn, affects the conformation and function of various ion channels and receptors, thereby activating a wide range of downstream signaling pathways [108].

The plasma membrane of endothelial cells is linked to the actin cytoskeleton, and these connections are an important part of the mechanism of mechanical transduction [109,110]. Indeed, the interaction of hemodynamic forces with the cell begins with the plasma membrane. Therefore, the mechanisms involved in the transformation of cellular strain into molecular signaling pathways mediating this mechanical transduction are of great importance [109,110,111].

Experimental evidence suggests that changes in shear stress affect the ordering of lipids in endothelial cell plasma membranes. This entails changes in some physical properties of the plasma membrane, such as fluidity and viscosity [112,113,114,115]. It was shown that a shear stress of 10 dyne/cm^2^ in laminar flow led to a rapid decrease in lipid order of the plasma membrane, with the most pronounced changes in the ordered phases [116]. This corresponded to the transition of caveolae to a disordered state [116]. Importantly, changes in lipid order depend on shear stress intensity and are reversible [38]. In addition, a similar decrease in lipid order was observed in artificial membranes that were exposed to shear stress, highlighting the physical basis of this mechanism [116].

It is assumed that the nature of the physical impact on the membrane differently affects the ordering of lipids in the plasma membrane, which allows endothelial cells to distinguish between shear stress and stretching [117]. Stretching leads to an increase in cholesterol content in the plasma membrane, which corresponds to a transition from a liquid-unordered phase to a liquid-ordered phase in some areas, as well as a decrease in membrane fluidity [117]. In turn, shear stress is characterized by a decrease in cholesterol levels, which decreases lipid order and increases membrane fluidity [117]. A decrease in lipid order during the phase transition of the liquid-ordered phase into the liquid disordered phase under the influence of shear stress, leads to a decrease in the number of caveolae [113,116].

Increasing evidence suggests that lipid rafts are important platforms for membrane-cytoskeleton coupling. The regulatory phospholipid PIP2 is involved in the regulation of cytoskeleton-membrane coupling [16,118,119]. Cholesterol has been shown to be involved in the regulation of this connection [16]. Changes in membrane cholesterol levels affect membrane and cytoskeleton interactions. Although cholesterol enrichment of plasma membranes leads to a decrease in their fluidity and viscosity [19], cholesterol enrichment of endothelial cell membranes causes a decrease in surface viscosity [16,119] and weakening of membrane-cytoskeleton adhesion [118,119,120,121]. Cholesterol depletion, although resulting in impaired lipid rafts, increases endothelial cell rigidity, which is associated with increased plasma membrane attachment to the actin cytoskeleton [16,118,119]. OxLDL has been shown to deplete cholesterol from caveolae by acting as a cholesterol acceptor [122]. The oxLDL-induced depletion of cholesterol levels in the caveolae causes eNOS to leave the caveolae and inhibits enzyme activation [122]. Reduced membrane and cytoskeletal adhesion may have important consequences for the development of atherosclerosis [119]. Indeed, endothelial cell stiffness was shown to be increased in atherosclerosis-prone areas that were characterized by impaired flow patterns compared with arterial sections, where higher shear stress values and more uniform unidirectional blood flow were observed [123].

Experiments with exposure of human umbilical vein endothelial cells to reduced shear stress showed reorganization of the actin cytoskeleton with a decrease in the number of stress fibers and actin recruited to the peripheral cell band, indicating an important relationship between the physical parameters of blood flow and endothelial cell phenotype [124].

As noted above, the degree of unsaturated fatty acids in cell membrane lipids determines membrane fluidity. It is of interest to learn that the expression of stearoyl-CoA desaturase-1 (SCD1), an enzyme that biosynthesizes monounsaturated fatty acids, is associated with hemodynamics in endothelial cells. The main product of SCD1 is oleic acid, which is formed by desaturation of stearic acid. Laminar flow increases the expression of SCD1, which may act as an important mechanism to regulate plasma membrane fluidity [125]. Laminar flow-induced expression of SCD1 can lead to an increase in membrane fluidity and modulate endothelial cell responses to various proatherogenic stimuli. Induction of stearoyl-CoA desaturase protects human arterial endothelial cells from palmitate-induced lipotoxicity, cellular apoptosis, and expression of the inflammatory cytokines IL-6 and IL-8 [126]. Conversion of saturated stearic acid to unsaturated oleic acid may have an atheroprotective effect by displacing saturated fatty acids from cell membrane phospholipids with subsequent modulation of gene expression of molecules involved in monocyte recruitment [127].

The data on the involvement of VEGFR2 in the cross-linking between chemoreception and mechanoreception in endothelial cells seem interesting. VEGFR2 localizes in lipid rafts, where cholesterol stabilizes the dimeric state of VEGFR2 and can affect its signaling pathway [128,129]. Both decreased and increased amounts of cholesterol in the plasma membrane can affect the structure of lipid rafts and influence VEGFR2 signaling [128]. Disruption of lipid rafts leads to significantly less receptor activation in response to VEGF exposure [129]. Restoration of normal cholesterol content in lipid rafts of endothelial cells stabilizes the dimeric state of VEGFR2 and angiogenesis [128].

Laminar blood flow with a shear stress of 12 dyne/cm^2^ affecting membrane lipid organization is able to activate VEGFR2 and its signaling pathways, including PI3K-Akt-eNOS, in a ligand-independent manner [130,131]. Thus, VEGFR2 can be regarded as a sensor capable of integrating chemical and mechanical information [132]. In this case, blood flow and VEGF-A act together to promote endothelial cell alignment and polarity. The central role of VEGFR2 in these processes is supported by evidence that inhibition of VEGFR2 results in the loss of influence of both shear stress and VEGF-A on endothelial cell alignment and polarity [132].

Nitric oxide is considered a key player in vascular hemodynamics. This molecule, produced by the endothelium via a specific nitric oxide synthase isoform, has a wide range of physiological functions. In addition to targeting vascular smooth muscle cells, through the relaxation of which NO exerts vasodilatory properties, nitric oxide performs a number of other important physiological functions. Reduced availability of endothelial NO is thought to be associated with endothelial dysfunction and the development of atherosclerosis. Endothelial cells are key participants in the regulation of vascular hemodynamics through the production of NO via endothelial nitric oxide synthase (eNOS) [5]. eNOS has a complex system of regulation, including that associated with its membrane localization. Binding of caveolin-1 to eNOS is known to keep eNOS in an inactive conformation, which reduces NO production [133,134,135,136]. Mice without caveolin-1 have no caveolae and show increased eNOS activity [28]. Shear stress increases eNOS transcription through several mechanisms [137]. Hemodynamic forces resulting from increased blood flow release eNOS from its inhibitory binding to caveolin and increase its binding to calmodulin, which causes eNOS dissociation from caveolin for flow-regulated NO production [138,139].

eNOS is known to be regulated by posttranslational modification, including phosphorylation, nitrosylation, and acylation. The localization of eNOS in caveolae depends on its myristoylation [49]. In addition, eNOS must be palmitoylated for localization in caveolae [56,140,141,142,143,144,145]. Myristoylation of eNOS is a prerequisite for the enzyme to undergo palmitoylation [146]. Myristoylation and palmitoylation give eNOS three acyl anchors that firmly attach it to caveolae [49]. Disruption of eNOS palmitoylation leads to decreased nitric oxide production [56,147]. Palmitoylation of eNOS can be partially controlled by fatty acid availability. Deletion of fatty acid synthase impairs eNOS palmitoylation and NO production by endothelial cells [56,147]. Mice with endothelial inactivation of FAS (FASTie mice) showed decreased membrane eNOS activity, which corresponded to a proinflammatory state in mice characterized by increased vascular permeability and the presence of endothelial inflammatory markers [148].

G-protein-coupled receptors (GPCRs) are the largest family of integral membrane proteins that control multiple cellular functions when binding to a variety of extracellular ligands. Due to their architecture of seven transmembrane domains, GPCRs cross the membrane seven times and are therefore closely related to their membrane environment [149]. Membrane cholesterol has been shown to be a critical modulator of GPCR organization, oligomerization, and function. A possible mechanism underlying modulation of GPCR function by cholesterol could be the specific interaction of GPCRs with membrane cholesterol, or cholesterol-induced changes in global bilayer properties, or a combination of both mechanisms [149].

It has been shown that G-proteins can be activated by shear stress [150,151], then suggests a link between GPCRs and mechanoreception. It is known that shear stress can directly affect the physical properties of the membrane, which can regulate the function of membrane proteins [152,153]. It is suggested that shear stress can be transmitted through the lipid bilayer to directly activate heterotrimeric G-proteins on the cytosolic surface of the plasma membrane [150].

Thus, the action of physical factors on endothelial cells is a significant factor in vascular biology, many aspects of which are still unknown. A growing body of evidence suggests that endothelial cell mechanoreception is closely related to endothelial cell plasma membrane function. This mechanoreception is associated with numerous signaling pathways affecting both endothelial cells themselves and other cells in the vascular wall through the production of biologically active molecules.

## 5. The Importance of ABC Transporters

ATP-binding Cassette (ABC) transporters are a large family of proteins that are involved in the transport of various substrates across cell membranes [154]. In humans, 48 transporters of this family are known, divided on the basis of their structural features into 7 subfamilies (ABCA-ABCG) [155]. Currently, the role of several ABC transporters in the pathogenesis of atherosclerosis is well known [156]. To the greatest extent, it concerns representatives of subfamilies ABCA and ABCG due to their participation in lipid transport. At the same time, members of the ABCB and ABCC subfamilies are involved in the mechanisms of multidrug resistance, which is also of significant clinical interest [154,157].

ABCA1 is well known for its atheroprotective role through its involvement in reverse cholesterol transport, a process in which cholesterol is exported from cells to an extracellular acceptor to form HDL [158]. Macrophages release excess cholesterol in this way, which is considered to be one of the key functions in providing cholesterol homeostasis [159]. ABCA1 is localized in the plasma membrane of cells but is able to recirculate between the plasma membrane and cellular organelles, which provides its transport function [160]. Through its function, ABCA1 affects cellular cholesterol content and is also associated with several plasma membrane functions, including phagocytosis, endocytosis, and microvesiculation [161,162,163,164,165].

ABCA1 is an integral protein with multiple transmembrane domains and can affect membrane lateral organization. ABCA1 is localized in non-raft domains and can disrupt lipid rafts and redistribute cholesterol to non-raft domains [161,166,167,168]. At the same time, ABCA1 also disrupts caveolae formation and can move phospholipids and cholesterol from the inner bilayer to the outer bilayer, which leads to membrane destabilization [161]. Thus, ABCA1 promotes redistribution of cholesterol into cell surface domains accessible to removal by apolipoproteins [169]. This is because ApoA-I preferentially accepts cholesterol from loosely packed non-raft microdomains [161,166,170]. The regulation of cholesterol content in the plasma membrane by ABCA1 ensures lipid raft stability, and may participate in signal transduction by VEGFR2 [171].

ABCA1 is known to be involved in the formation and maintenance of membrane lipid asymmetry by moving, for example, phosphatidylserine from the inner leaflet of the plasma membrane to the outer leaflet [172].

An important effect of the lipid-transporting function of ABCA1 is its role in reducing the surface presentation of TLR4 and recruiting the receptor into lipid rafts, which has anti-inflammatory effects [168,173]. It has been shown that ABCA1 expression in macrophages can protect against atherosclerosis through reduction in cholesterol in macrophage plasma membrane and lipid raft content, which suppresses proinflammatory MyD88-dependent signaling pathways [174]. ABCA1 deficiency in macrophages leads to increased translocation or recruitment of MyD88-dependent TLR2, TLR 4, TLR 7, and TLR9 to lipid rafts. This results in enhanced downstream signaling and increased production of proinflammatory cytokines [168]. In addition, ABCA1 enhances TLR4-stimulated interleukin-10 (IL-10) secretion through protein kinase A (PKA) activation [175]. ABCA1-mediated PKA activation has been shown to promote an M2-like inflammatory response by bone marrow-derived ABCA1+/+ macrophages. In contrast, cholesterol enrichment suppresses PKA activity and promotes an M1-like inflammatory response [175].

Thus, ABCA1 is an important participant in the lipid-transport function of cells, due to which the transporter is involved in the regulation of inflammation, which is of great importance in the prevention of atherosclerosis [172].

Another member of this subfamily, ABCA7, is thought to be involved in phagocytosis [176,177,178]. ABCA7 is expressed in macrophages, where it can be localized in the plasma membrane, or intracellularly [176,179,180,181,182,183,184]. The CED-7 protein in the nematode *Caenorhabditis elegans*, which functions in phagocytic and apoptotic cells during phagocytosis and is required for clustering CED-1, a transmembrane receptor that initiates uptake signals, has a high similarity in amino acid sequences to ABCA7. In this case, CED-7 carries out the exposure of phospholipid ligands on the surface of apoptotic cells [177].

ABCB1, well known for its role in multidrug resistance through its ability to efflux a variety of molecules with different chemical structures, also has other physiologically relevant functions, such as participation in lipid transport [185,186,187] by moving lipids from the inside to the outside of the cell plasma membrane [71,186]. ABCB1 is also involved in cholesterol transport [188]. At the same time, the cholesterol content in the plasma membrane can affect the functional activity of the transporter [189,190,191,192], which can be related to the direct interaction of ABCB1 with lipid molecules [71,186,193].

ABCG1 is also involved in the export of cholesterol from peripheral cells and the saturation of HDL [194,195,196,197]. ABCG1 is also involved in the regulation of inflammation [198]. ABCG1 has been shown to be involved in macrophage polarization. ABCG1 deficiency contributes to proinflammatory M1 polarization of human macrophages. Abcg1^−/−^ macrophages have been shown to engulf apoptotic cells but are unable to remove cholesterol derived from these apoptotic cells to the necessary extent, which is consistent with the propensity of Abcg1^−/−^ alveolar macrophages for apoptosis [199,200].

Thus, ABC transporters may be involved in the regulation of plasma membrane composition and function, and these processes may be impaired in atherosclerosis.

## 6. The Significance of PUFAs

A growing body of clinical evidence is increasing interest in polyunsaturated fatty acids derived from marine fish in the prevention of atherosclerosis. ω-3 PUFAs are involved in many physiological processes in the vascular wall. They are a substrate for the synthesis of specialized pro-resolving mediators and can also act on innate immune system receptors themselves, demonstrating anti-inflammatory properties. In addition, fatty acids can be incorporated into membrane phospholipids, thus participating in the maintenance of the biophysical properties of plasma membranes. The fatty acid composition of phospholipids provides, on the one hand, optimal fluidity, which is required for the necessary conformational changes of proteins when performing their functions, and, on the other hand, provides sufficient viscosity, which is required for the membrane localization of proteins [39,201]. Phospholipid tails of saturated fatty acids provide an ordered lipid organization, whereas in contrast, phospholipids containing polyunsaturated fatty acids are less disordered. When incorporated into membrane phospholipids, ω-3 PUFAs displace cholesterol, which affects the lipid ordering of microdomains [202]. This is due to the structural features of PUFAs, whose acyl chains have many rapidly changing conformations that repel the rigid steroidal part of cholesterol, which affects the lipid ordering of the membrane [202,203].

In this regard, the incorporation of ω-3 PUFA into phospholipids of plasma membranes can significantly affect the organization of their molecular architecture as well as lipid-protein interactions and the functions of membrane proteins [202]. Eicosapentaenoic acid affects lipid composition in caveolae, promoting eNOS translocation from caveolae to soluble fractions, which leads to stimulation of NO production [204]. In turn, docosahexaenoic acid affects caveolae not only by changing their lipid composition but also through the distribution of key structural proteins, such as caveolin-1, and also promotes eNOS displacement from caveolae [205]. Treatment of cells with docosahexaenoic acid has been shown to increase eNOS activity [205].

Interestingly, PUFAs can act differently on the lipid organization of membranes, which is determined by the chain length of the fatty acids and the degree of their unsaturation. Eicosapentaenoic acid prefers a disordered region; whereas docosahexaenoic acid can penetrate lipid rafts, affecting their structural organization [206]. Eicosapentaenoic acid inhibits the formation of cholesterol domains, reduces membrane fluidity, and normalizes bilayer width in atherosclerotic model membranes [207]. At the same time, docosahexaenoic acid induced the formation of cholesterol domains and increased the fluidity of the model membrane without affecting the membrane bilayer width [207]. Compared to eicosapentaenoic acid, exposure to docosahexaenoic acid can increase the fluidity of the plasma membrane of endothelial cells to a greater extent. Thus, eicosapentaenoic acid and docosahexaenoic acid can counteract each other’s physiological effects in individual tissues to a certain extent [206]. These effects may be related to the fact that docosahexaenoic acid is the most unsaturated and one of the longest ω3 PUFAs, due to which it has a significant influence on the biophysical properties of plasma membranes [203].

These effects on membrane biophysical properties and other physiological functions make PUFAs an interesting topic for clinical research.

## 7. Conclusions

**Figure 2:**
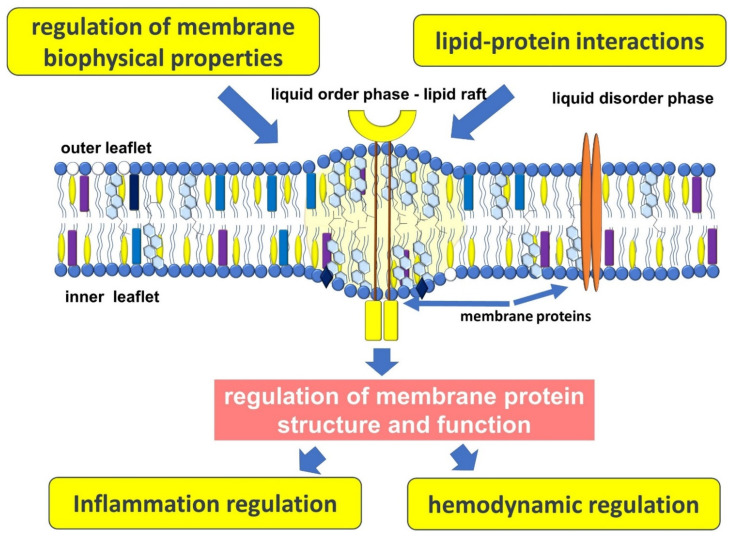
Plasma membrane function in atherogenesis.

Atherosclerosis is an important medical and social problem, the keys to understanding the many links in the pathogenesis of which are still largely unknown to clinicians and researchers. Atherosclerosis develops over many years and is a complex chain of events, many links of which are closely linked. It is believed that disorders of lipid metabolism are an important cornerstone in the pathogenesis of atherosclerosis. This is due to the multifaceted role of lipids, not only as energy substrates or a structural component of cells, but as an important participant in many processes occurring in the vascular wall (Figure 2).

A growing body of evidence confirms the role of the plasma membrane not only as a structural unit of the cell, which delimits it from the surrounding space, but also as an important platform on which many important processes take place. Thus, there is an increasing understanding that the plasma membrane can be considered as a dynamic system regulating many cellular functions, the disturbance of which is associated with atherogenesis.

## Data Availability

Not applicable.
